# When to Hold That Thought: An Experimental Study Showing Reduced Inhibition of Pre-trained Associations in Schizophrenia

**DOI:** 10.1371/journal.pone.0042175

**Published:** 2012-07-30

**Authors:** Zhimin He, Helen J. Cassaday, S. Bert G. Park, Charlotte Bonardi

**Affiliations:** 1 Division of Psychiatry, School of Psychology, University of Nottingham, Nottingham, United Kingdom; 2 Division of Psychiatry, School of Community Health Sciences, University of Nottingham, Nottingham, United Kingdom; Ecole Normale Supérieure, France

## Abstract

Schizophrenia encompasses a wide variety of cognitive dysfunctions, a number of which can be understood as deficits of inhibition. To date, no research has examined ‘conditioned inhibition’ in schizophrenia - the ability of a stimulus that signals the absence of an expected outcome to counteract the conditioned response produced by a signal for that outcome (a conditioned excitor). A computer-based task was used to measure conditioned excitation and inhibition in the same discrimination procedure, in 25 patients with a confirmed diagnosis of schizophrenia and a community-based comparison sample. Conditioned inhibition was measured by a ratio score, which compared the degree to which the inhibitory stimulus and a neutral control stimulus reduced conditioned responding to the excitatory cue: the lower the ratio, the greater the inhibitory learning. At test the ratios were 0.45 and 0.39 for patient and control groups respectively, and the relevant interaction term of the ANOVA confirmed that the degree of inhibition was reduced in the patient group, with an effect size of *r* = 0.28.

These results demonstrate for the first time that inhibitory learning is impaired in schizophrenia. Such an impairment provides an attractive framework for the interpretation of the positive symptoms of schizophrenia. However, we were unable to demonstrate any relationship between the level of conditioned inhibition and medication. Similarly, in the present study it must be emphasised that the available data did not demonstrate any relationship between individual variation in inhibitory learning and the level of positive symptoms as measured by the PANSS. In fact inhibitory learning impairment was relatively greater in participants with a predominantly negative symptom profile and their excitatory learning was also reduced. Accordingly the next step will be to investigate such relationships in a larger sample with *a priori* defined sub-groups displaying predominantly positive versus predominantly negative symptoms.

## Introduction

Cognitive dysfunction is a definitive aspect of schizophrenia [1], [2], and the information-processing abnormalities associated with this condition are diverse. However, one emergent theme is that many of these impairments can be broadly understood as varieties of inhibition deficit [3], [4], [5], [6], [7], [8], [9], [10], [11], [12]. Yet it would be simplistic to describe schizophrenia as a deficit in inhibition, because the ‘inhibitory’ processes supposedly affected are very diverse. For example, a disruption in prepulse inhibition [13] - the reduction in the *unlearned* startle response produced by a weaker version of the later presented startle stimulus - has been reported in schizophrenic populations [11], [14], [15], [16], [17], [18], [19]. There have also been reports of a deficit in latent inhibition (LI) [20], which is the slowed acquisition of a *learned* (or conditioned) response to a conditioned stimulus (CS), which signals a stimulus of intrinsic affective value (an unconditioned stimulus, or US). LI results if the conditioned stimulus is pre-exposed prior to the conditioning treatment [3], [9], [21], [22], [23], [24].

Another example of inhibition of learned responding is conditioned inhibition (CI). A conditioned inhibitor is a stimulus which predicts that an otherwise expected outcome will not occur [25], [26]. For example, if stimulus A signals the US when presented alone, but after a compound of A with a further stimulus, B, the US is omitted (AB−), B is termed a conditioned inhibitor [25]. This is evident in B's resultant ability to suppress the conditioned responding produced by other signals for that same US. CS pre-exposure retards acquisition of CI just as it retards CS→US (or excitatory) learning [27], [28], indicating the distinction between CI and LI. Indeed LI has often been interpreted as a loss of attention to the pre-exposed cue which disrupts both excitatory and inhibitory learning [29]. There are reasons for expecting that CI will also be disrupted in schizophrenia: CI is reduced in participants with high schizotypy [30], and in animal studies the dopaminergic system has been identified as a key substrate that mediates CI [31], [32]. This was the starting point for the present investigation.

A deficit in CI could help explain some of the cognitive symptoms of schizophrenia. Paradigms such as LI and CI were developed in studies with animals, and are grounded in classical conditioning theory, which describes learning about signals for motivationally significant outcomes that elicit involuntary, unlearned responses comprising behavioural, cognitive and affective components. Some of these components will also be present in the conditioned response to the CS that signals that outcome. These conditioned responses are involuntary, and so CI may be understood without recourse to higher cognitive constructs. Yet an inhibitor can be regarded as potentially inhibiting not only the behavioural responses elicited by the CS, but also the affective and cognitive responses that are associated with it - meaning that it can affect behaviour at a number of levels. Moreover, although the behavioural changes directly attributable to the CS or the inhibitor follow more or less immediately [33], the internal state associated with schizophrenia might conceivably act as an internal context, as has been proposed for depression [34], [35], in which a failure to learn about conditioned inhibitors would be embedded. A failure to inhibit various associations could thus be activated by this internal state, and in this manner contribute to symptoms of schizophrenia, such as sensory flooding and delusions. For example, in a healthy subject the chance pairing of a mundane object with an emotionally significant event will not influence subsequent behaviour, because on subsequent occasions they will learn that the expected motivationally significant event no longer occurs - via the inhibitory learning process. If this learning is impaired in the schizophrenia sufferer, then the events of everyday life will remain significant and continue to demand attention, resulting in aberrant behaviour. Similarly, patients experience delusions of reference when they perceive stimuli provided by exposure to the media or being in some public place as pertaining specifically to them. Recent functional magnetic resonance imaging studies show that both patients with schizophrenia and healthy controls with experimentally induced self-referential ideation (using individually specific information taken from an interview conducted some weeks earlier), display characteristic patterns of brain activation in cortical midline structures, as well as in interconnected midbrain dopaminergic regions implicated in schizophrenia and CI. In contrast, normal participants presented with nonpersonalised experimental materials are able to inhibit associations with their current circumstances and interests [12].

In summary, there are both empirical and theoretical grounds for hypothesising that conditioned inhibitory learning might be impaired in participants with schizophrenia. The present study examined whether this was in fact the case.

## Methods

### Objectives

The primary objective of the present study was to test the hypothesis that CI would be impaired in participants with schizophrenia. We also sought to establish whether the level of CI shown was systematically related to symptom severity.

### Participants

The experiment was conducted on 25 patients from three different adult mental health residential units in the city of Nottingham, UK. Diagnoses of schizophrenia met the International Classification of Diseases [36] criteria for schizophrenia, in the absence of comorbid mental conditions. Patients from two of the three units had a formal psychiatric assessment of symptom severity using the KGV scale [37]. All 25 patient participants completed the computer task. Twenty of these also completed the Positive and Negative Syndrome Scale (PANSS) interview [38] to assess their current (or recent) symptoms, 11 on the same day as the CI task. In total 9 participants did not complete the PANSS on the same day as the behavioural test but were willing to do so. They were interviewed at the earliest mutually convenient which turned out to be within 3–7 weeks. The remaining 5 participants were unwilling to complete the PANSS interview in addition to the behavioural test. Table 1 shows the summary PANSS scores. Participants were under a variety of antipsychotic medication regimes. Calculation of the chlorpromazine (CPZ) equivalent was based on: 100 mg/day CPZ = 5 mg/day olanzapine, 100 mg/day clozapine, 200 mg/day sulpiride, 1 mg/day risperidone [39], [40], [41], [42], [43].

**Table 1:** Summary details of the patients' PANSS scores.

	PANSS Positive	PANSS Negative	PANSS General	PANSS Total
**Mean (SD)**	14.10 (4.45)	18.65 (8.36)	28.10 (5.86)	60.85 (12.62)
**Range**	7–21	8–36	16–37	36–79

*Note:* Mean and standard deviation (SD) of patients' (n = 20) scores on the different sub-scales of the PANSS, together with the minimum and maximum (Range) of scores on each sub-scale.

The controls were a community-based sample of 25 participants living in the same county, matched as far as possible on age, ethnicity and educational status (see Table 2). None reported or showed any indication of mental illness or substance abuse. All were tested under comparable, quiet environmental conditions by ZH. All 25 control participants completed the computer task.

**Table 2:** Summary details of the final sample of participants.

	Schizophrenic patients (n = 25)	Control participants (n = 25)
Age (years)	30.64	31.20
Age range (years)	20–41	19–48
Gender (N = male/female)	18/7	18/7
Education range (years)	11–15*	11–14*
Ethnicity	24 White and 1 Black	24 White and 1 Black

*Note:* * In the UK, the number of years in education required to achieve A level is 14. The patient records did not give full details of level of education, so patient participants were asked whether they had attended university (just one who had dropped out in year 1, hence no undergraduate participants were included in the matched control group). Based on the available data (N = 25 for controls and N = 21 for patients) the median level of education was 12 years for both patient and control participants, and on a Mann-Whitney U test there was no significant difference between the patient and control groups, *p* = 0.088.

### Ethics

The study was approved by UK NHS Research Ethics (Derbyshire Research Ethics Committee, reference No. 08/H0401/65, September 2008), and by the University of Nottingham, School of Psychology Ethics Committee. As an inconvenience allowance, control participants received £5, and schizophrenic participants £10. Before the task, each participant was required to read the information sheet and sign a consent form. Patients' capacity to give consent was based on the judgement of the clinical staff who had duty of care at their residential unit on that day. If a potential participant was deemed unable to give informed consent, they were not approached to take part in the study.

### Design

The design of the experiment is shown in Table 3. There were three stages: (1) pre-experimental, (2) excitatory and inhibitory training, and (3) test [44].

**Table 3:** The design of the experiment.

Pre-experimental stage		(a) Excitatory training stage		(b) Inhibitory training stage		Test	
CSs	No. trials	CSs & USs	No. trials	CSs & USs	No. trials	CSs	No. trials
A	2	A+	12	AZ+	8	A	2
C	2	U−	12	AP−	12	C	2
AZ	2	V−	12	BX−	12	AZ	2
AP	2	C+	12	CY+	8	AP	2
BX	2					BX	2
CY	2					CY	2
CP	2					CP	4
CX	2					CX	4

*Note:* Letters denote the 9 conditioned stimuli (pictures of Lego blocks); the identities of which were counterbalanced (see Table 4). With respect to US presentations that immediately followed CS presentations during the training stages, ‘+’ represents a positive IAPS picture and ‘−’ a neutral IAPS picture; see text for the identities of the IAPS pictures which served as positive and neutral stimuli.

In the pre-experimental stage participants rated the neutral stimuli and stimulus compounds (A, C, AZ, AP, BX, CY, CP and CX) which were to serve as CSs in the subsequent stages, to allow control for preexisting biases.

The training stage comprised excitatory training, followed by inhibitory training. During the excitatory training phase four individual CSs were paired with either reinforcement (a positive picture, on A+ and C+ trials), or nonreinforcement (a neutral picture, on U− and V− trials); the difference in learning about reinforced and nonreinforced cues provided a measure of simple excitatory learning.

During the second, inhibitory training phase, the CS compound AZ signalled reinforcement (AZ+), whereas a second compound, AP, signalled nonreinforcement (AP−); P thus signalled the absence of the reinforcement predicted by A, and established P as a conditioned inhibitor. Two additional compounds, CY and BX, were reinforced and non-reinforced respectively (CY+, BX−); X was the control stimulus for the test that followed.

The final, test phase was designed to confirm that P was a conditioned inhibitor, by examining whether it would suppress responding to the excitatory C [26]. X, the control stimulus, differed from P only in that it had not signalled the absence of reinforcement, and so should not have acquired inhibitory properties. The critical comparison was thus between CP and CX. If CP was rated less positive than CX, this suggested that P was more effective in counteracting the ability of C to predict a nice picture, and was thus evidence that P was inhibitory.

### Stimuli

**Figure 1:**
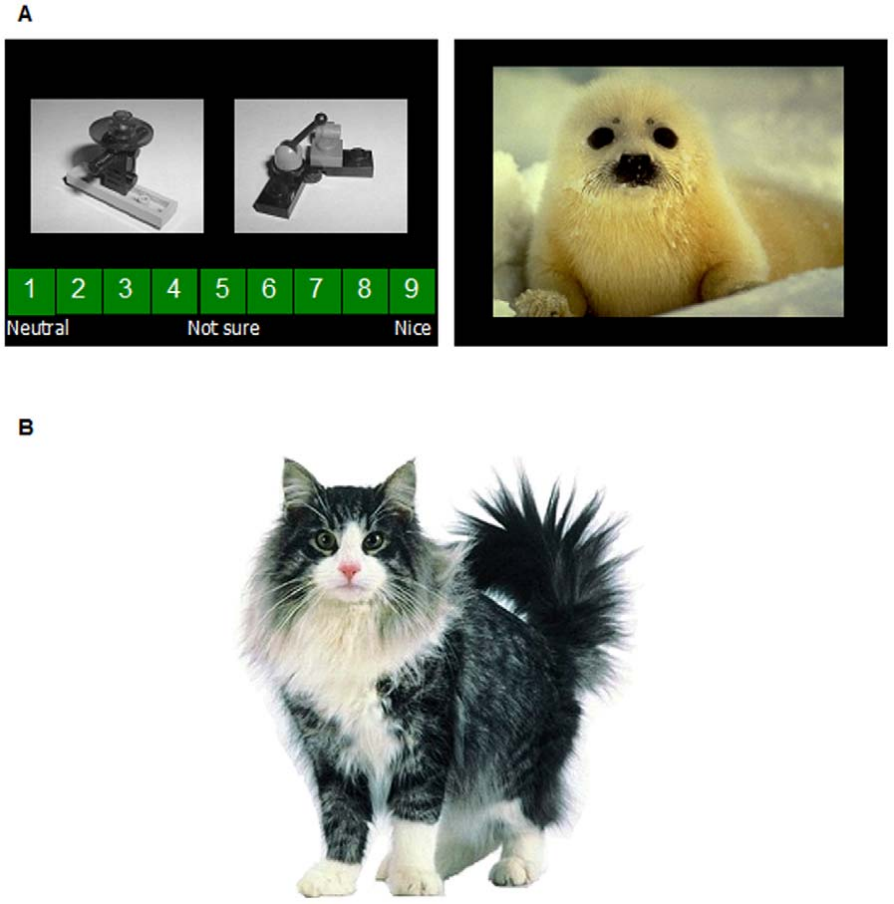
The rating scale and an example of a CS compound (top left panel), a positive US (top right panel), and the cat Mogwai (lower panel).

Nine Lego block pictures were used as CSs. P and X were counterbalanced, as were A and B, and C and V (see Table 4). The USs were images from the International Affective Picture System (IAPS) [45], a set of images standardised on the dimensions of valence and arousal from 1 to 9, 1 representing a low and 9 a high rating. The USs comprised 10 positive and 10 neutral pictures with mean valences (SD, range) positive = 7.89 (0.27, 7.56–8.28) and neutral = 4.94 (0.08, 4.86–5.08); mean arousal ratings (SD, range) positive = 4.86 (1.03, 3.08–6.73) and neutral = 2.79 (0.54, 1.72–3.46). The codes of the IAPS images which served as the positive USs were 1440, 1610, 1750, 1920, 8370, 8380, 2040, 2154, 2160 and 8496; those serving as negative pictures were images 2393, 2396, 2512, 2890, 7006, 7055, 7175, 7185, 7187 and 6150 [44]. Positive USs were presented on ‘reinforced’ trials and neutral USs on ‘non-reinforced’ trials. The measure of conditioning was a rating of what kind of picture the participant predicted would follow presentation of the CS, ranging from 1 (neutral) to 9 (positive), with a rating of 5 (‘not sure’, see Figure 1) intended to reflect uncertainty as to the following outcome; an average was calculated for each particular CS or CS combination in each phase.

**Table 4:** The identity of the various Lego blocks (I–IX) that served as the experimental stimuli in the eight counterbalanced subgroups.

Counterbalanced Group	Conditioned stimuli and identity of Lego block								
	A	B	C	P	X	Y	Z	U	V
1	I	II	III	IV	V	VI	VII	VIII	IX
2	I	II	III	V	IV	VI	VII	VIII	IX
3	II	I	III	IV	V	VI	VII	VIII	IX
4	II	I	III	V	IV	VI	VII	VIII	IX
5	I	II	IX	IV	V	VI	VII	VIII	III
6	I	II	IX	V	IV	VI	VII	VIII	III
7	II	I	IX	IV	V	VI	VII	VIII	III
8	II	I	IX	V	IV	VI	VII	VIII	III

### Statistical Analysis

A summary measure of excitatory learning was provided by the ratio of the mean ratings of the reinforced C and nonreinforced V, i.e. C/(C+V) from all trials of the excitatory training stage; greater C/(C+V) scores indicated greater excitatory learning. An *a priori* exclusion criterion (C/(C+V) = <.5) was applied to excitatory training performance, as a result of which nine participants (five patients and four controls) were excluded as being unable to learn the basic task. A summary measure of CI was provided by the ratio of the mean ratings of CP and CX, i.e. CP/(CP+CX); the lower this ratio, the greater the inhibitory learning. Prior to statistical analysis, the ratio measures were subjected to an arcsine root transformation [46]. Statistical analyses were by mixed design analysis of variance (ANOVA). Significant interactions were explored with simple main effects analysis using the pooled error term for between subjects contrasts. Planned comparisons of the assessment score data were by t-test. The measure of effect size given for mixed design ANOVAs was Pearson's correlation coefficient, *r*; following Field [47] *r* was calculated only for main effects with two levels and specific contrasts. Correlational analyses (Pearson's) were used to examine the relationship between learning scores and (1) symptom profile (measured by PANSS) and (2) antipsychotic medication dosage. For *r* values suggesting correlations at or close to statistical reliability we also report the coefficient of determination (*r^2^*) in order to consider the proportion of the variance explained. All statistics were performed with SPSS, apart from simple main effects analysis which was performed with Experstat.

### Procedure

The task instructions were that a cat ‘Mogwai’ would bring participants either a positive picture or a neutral, boring picture, depending on what kind of Lego blocks she found in her basket. Participants were told that they would be asked to guess, or predict, what kind of picture would follow presentation of the Lego blocks, using a rating scale from 1 (neutral) to 9 (positive), with a rating of 5 (‘not sure’, see Figure 1) intended to reflect uncertainty as to the following outcome. Reminder instructions were presented on-screen at each stage of the procedure.

Before the first phase participants were shown some representative US pictures, and also CS pictures with the rating scale, on 4.5×6 cm cards, and the rating procedure was explained; these pictures were not used in the experiment. Participants were told that the session, comprising three stages, would last about 20 minutes, and they were welcomed to ask questions.

### Pre-experimental stage

Participants were first instructed that they must guess what kind of picture the cat might bring based on the Lego blocks presented, although no pictures would follow. A CS was presented, after which participants clicked on a number button to guess the US valence; the next CS presentation followed immediately. There were 16 stimulus presentations, two of each of the following: A, C, AZ, AP, BX, CY, CP and CX (see Table 3). Throughout the experiment CS presentations were counterbalanced for right/left position on the screen, and the various trial types were presented in a semi-random sequence (constrained by the total number of trials of a particular type in each stage).

### Training stages

At the start of the first training stage the participants were instructed that, as before, they must predict what kind of picture the cat might bring, based on the Lego block that was presented, and that they would then be shown the picture that the cat had brought. The excitatory training stage comprised 6 training blocks, each with two of the four kinds of trial, A+, U−, V− and C+. After the participant had rated the valence of the predicted US, a US, randomly selected from the pool of positive or neutral USs as appropriate, was presented for 1 s. The next trial followed after a 1 s gap, during which a picture of the cat Mogwai (around 6×6 cm) was presented on a white background. The inhibitory training stage followed directly after this stage and comprised 4 kinds of trial (AZ+, AP−, BX− and CY+) presented in two blocks. Each block comprised 4 presentations of each reinforced compound and 6 of each non-reinforced compound.

### Test stage

The test stage was identical to the pre-experimental stage, except that there were four presentations of each of the test compounds CP and CX.

Throughout the experiment, whenever participants asked questions or made comments they were asked to try to focus on the task and to try to remember or guess which outcome (positive or neutral picture) was predicted by the Lego blocks.

## Results

### Pre-experimental stage

The mean ratings of CP and CX were, respectively, 4.9 and 4.76 for the patient group and 5.28 and 4.42 for the control participants. ANOVA with stimulus (CP v. CX) and group (schizophrenic patients v. controls) revealed no pre-existing differences in ratings of the two critical compounds CP and CX, *F*(1,48) = 1.73, *p* = 0.19, or any effect of, or interaction with, group, *Fs*<1.

### Training stage 1: Excitatory training

**Figure 2:**
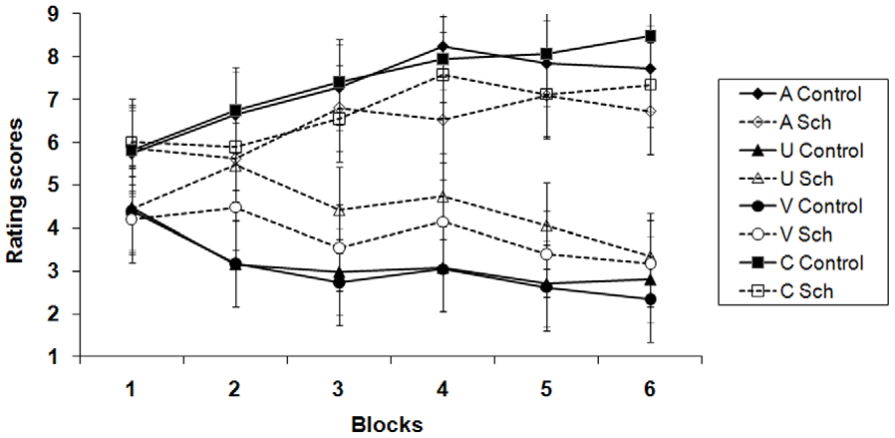
Group mean rating scores for A, U, V and C in the excitatory training stage. A rating of 9 reflected expectation of a positive image to follow, and 1 of a neutral image to follow; 5 indicated uncertainty as to the following outcome. Each block comprised two pairings of A and C with a positive picture, and two of U and V with a neutral picture. The error bars represent two standard errors of the mean.

The results of the initial training stage provided a measure of excitatory learning in the two groups; although both groups clearly learned the task, the patient group appeared to respond less on reinforced, and more on nonreinforced trials, than the control participants (Figure 2). ANOVA with group, discrimination (A+ versus U−, C+ versus V−), reinforcement and training block as factors revealed a significant interaction between reinforcement and diagnostic group, *F*(1,48) = 7.73, *p* = 0.008, *r* = 0.37; although both groups learned the discrimination, *F*(1,48) = 96.41 and 31.68 for control and patient groups respectively, *ps*<0.001, they differed on both reinforced, *F*(1,96) = 4.40, *p* = 0.04, and nonreinforced trials, *F*(1,96) = 7.95, *p* = 0.006. This suggests some degree of learning impairment in the patient group. This was confirmed by an analysis of the summary measure of excitation, C/C+V; the mean score was .64 for the patient group and .72 for the control group, and these values differed significantly, *F*(1,48) = 5.25 *p* = .026 *r* = .314. For additional details please see Supporting Information S1.

### Training stage 2: Inhibitory training

**Figure 3:**
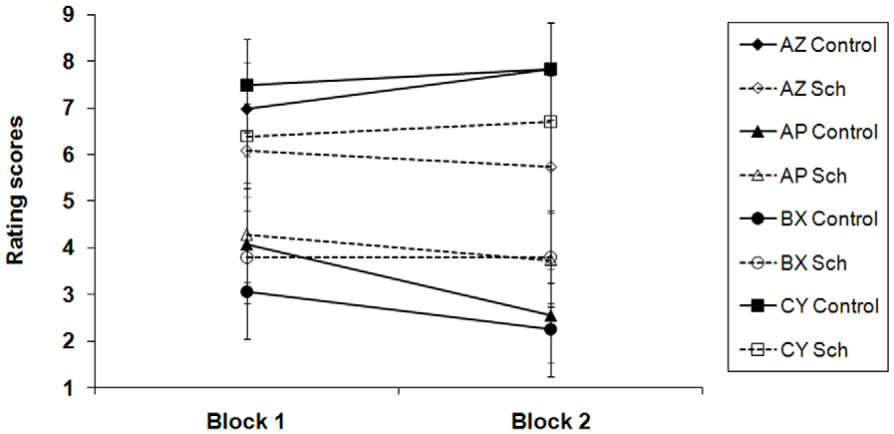
Group mean rating scores for AZ, AP, BX and CY in the inhibitory training stage. A rating of 9 reflected expectation of a positive image to follow, and 1 of a neutral image to follow; 5 indicated uncertainty as to the following outcome. Each block comprised four presentations of the stimulus compounds AZ and CY paired with a positive picture, and six of compounds AP and BX paired with a neutral picture. The error bars represent two standard errors of the mean.

During this stage participants were trained on the key discrimination between AZ+ and AP−, which was designed to turn P into a conditioned inhibitor; they were also required to discriminate between CY+ and BX−. Both groups learned these discriminations, but again the patient group showed slightly poorer performance (Figure 3).

ANOVA with group (patient v. control), discrimination (AZ+ v. AP− and CY+ v. BX−), reinforcement (reinforced or not) and training block (1–2) as factors revealed a significant interaction between reinforcement and group, *F*(1,48) = 11.08, *p* = 0.002 *r* = 0.43. Although both groups learned the two tasks, *F*(1,48) = 92.84 and 24.29 for patient and control groups respectively, *ps*<0.001, the groups differed on both reinforced and non-reinforced trials, *F*(1,96) = 9.99, *p* = 0.002, and *F*(1,96) = 5.01, *p* = 0.03. For additional details please see Supporting Information S1.

### Test stage

**Figure 4:**
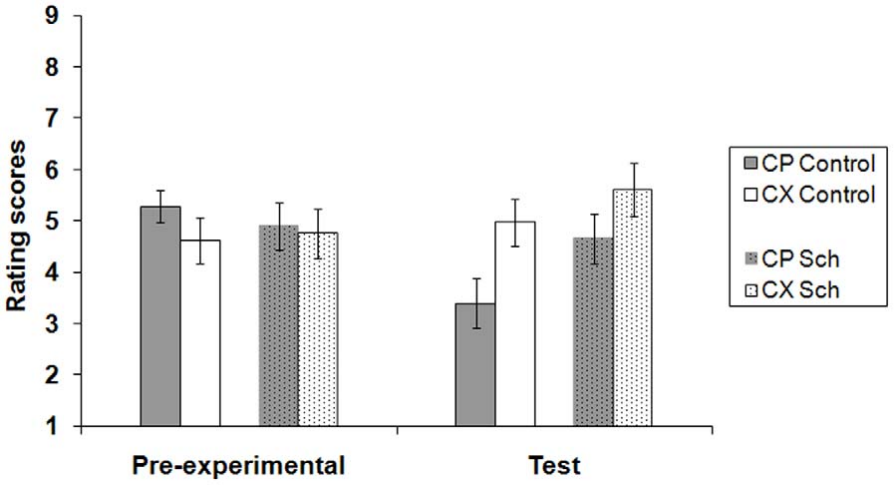
Group mean rating scores for C in compound with the inhibitor P and the control stimulus X in the pre-experimental and the test stages. A rating of 9 reflected expectation of a positive image to follow, and 1 of a neutral image to follow; 5 indicated uncertainty as to the following outcome. The error bars represent two standard errors of the mean.

It is clear from Figure 4 that, although during the pre-experimental stage both groups rated CP and CX similarly, during the test phase CP was rated lower than CX, suggesting P had become inhibitory. Critically, this effect seemed more marked in the control participants. To evaluate this the ratio CP/(CP+CX) was computed for both pre-experimental and test stages for each group. The resulting scores for the pre-experimental stage were 0.51 for the patient group and 0.56 for the controls; neither score differed from 0.5, *p* = 0.71 and 0.08 respectively, confirming that there were no pre-existing biases in responding to CX and CP. At test the ratios were 0.45 and 0.39 for patient and control groups respectively, and both differed from 0.5, *p* = 0.015 and 0.007 respectively, confirming that P had acquired inhibitory properties in both groups. Nonetheless the degree of inhibition appeared reduced in the patient group, and ANOVA with group and stage (pre-experimental and test) as factors confirmed this, revealing a significant interaction, *F*(1,48) = 4.05, *p* = 0.049, *r* = 0.28; the effect of stage was significant in the control group, *F*(1,48) = 18.54, *p* = 0.001, but not in the patients, *F*(1,48) = 2.13, *p* = 0.15.

ANOVA comparing group mean ratings of CX in both stages revealed no significant effect of group, stage, or interaction between the two factors, *F*<1, *F*(1,48) = 2.47, *p* = 0.12, and *F*<1, respectively, confirming that there were no group differences in responding to CX, the baseline against which the effect of P was evaluated.

### Differences by symptom profile

The summary measures of excitation (C/(C+V)) and inhibition (CP/(CP+CX)) did not correlate with the PANSS General score, *r*(20) = −0.14, *p* = 0.57, and *r*(20) = −0.29, *p* = 0.22, respectively. However, the summary inhibition measure correlated significantly with the PANSS Negative symptom scores, *r*(20) = 0.45, *p* = 0.05, accounting for approximately 20% of the variance, *r^2^* = 0.20, while the corresponding relationship with the summary excitatory measure was marginal but similar in magnitude, *r*(20) = −0.41, *p* = 0.07, accounting for approximately 17% of the variance, *r^2^* = 0.17. In contrast the PANSS Positive symptom scores correlated neither with inhibitory, *r*(20) = −0.16, *p* = 0.50, nor excitatory summary measures, *r*(20) = −0.05, *p* = 0.83. Therefore the relationship between symptom profile and performance on the summary learning measures at test was confined to a tendency to lower expressed CI on a background of similarly reduced excitatory learning in participants with a negative symptom profile.

### Differences by medication

There were no detectable differences by medication status: there was no correlation between dose, measured as the CPZ equivalent, and either inhibitory, *r*(21) = −0.32, *p* = 0.16, or excitatory learning scores, *r*(21) = −0.04, *p* = 0.86. Neither were there any differences in inhibitory or excitatory learning between the schizophrenic patients on typical and atypical antipsychotics, *t*(17) = 1.53, *p* = 0.15; *t*(17) = 0.68, *p* = 0.50, respectively.

## Discussion

The present study demonstrated that CI was impaired in schizophrenic compared to matched community control participants. While both groups responded similarly to the excitatory C in compound with the neutral X, the ability of the inhibitor P to inhibit responding to C was significantly reduced in the patient group. Aside from the fact that P, not X, had signalled the absence of the outcome, both stimuli had been trained identically – being non-reinforced in the same number of compound stimulus presentations. This difference cannot therefore be attributed to excitatory learning at the test stage, or nonspecific effects on performance, and is most readily interpreted as a deficit in CI. Schizophrenic participants were also less efficient at excitatory conditioning, responding less on reinforced trials and more on nonreinforced trials in the training stage than the control participants. However, this difference in excitatory learning did not compromise our demonstration of CI deficit. Participants who did not meet an *a priori* criterion for excitatory learning (5 patients and 4 controls) were excluded, and at test there was no group difference in responding to CX, the excitatory baseline against which the inhibitory effect of P was assessed.

Nor was there evidence that differences in either excitatory or inhibitory learning could be linked to medication. However, the patient participants with higher PANSS negative scores tended to show generally poorer excitatory and inhibitory learning.

### Limitations

These conclusions rely naturally on the adequacy of our control condition. For example, group differences in general intelligence or motivational factors cannot be ruled out, although control participants were, as far as possible, matched in terms of factors such as educational level and socio-economic status. Similarly, it was not possible to give the control participants a structured clinical interview to rule out mental illness or substance abuse - although behavioural differences were observed despite such potential confounding factors. Nor was it practicable for the experimenter to be blind to group membership, but the task was fully automated, minimising the possibility of experimenter effects. In addition most patients were medicated, although we did not detect any effects of medication on either excitatory or inhibitory learning, the numbers of participants in these analyses were necessarily small.

Five patients were unwilling to complete the PANSS assessment and a further 9 patients were unable to complete it on the same day as the behavioural test. This was an exploratory analysis intended to help identify the underlying mechanisms of any group differences (and the available data was limited). A modest relationship between CI and negative symptom score was demonstrated despite the relatively small sample size and differences in when the PANSS was administered. Excitatory learning was similarly reduced in those with more negative symptoms; thus CI was not selectively impaired in relation to negative symptoms.

Impaired associative learning is often reported in schizophrenia, in the control conditions of LI and blocking tasks [3], [48], raising the possibility that the attenuation of CI we observed is secondary to a more general impairment in excitatory associative learning. As a conditioned inhibitor signals the absence of an outcome predicted by an excitatory stimulus, if earlier learning about this excitatory stimulus is reduced, CI will be impaired. We cannot rule out this possibility on the basis of the present data. Nonetheless, attenuated CI in schizophrenia has not previously been demonstrated and - even if it is related an excitatory learning deficit at the training stage - will have effects on behaviour quite different to those produced by a pure excitatory learning impairment.

### Implications

Despite their inevitable inter-dependency, animal studies suggest that inhibitory and excitatory learning are dissociable [26], [49], meaning that distinct neural substrates could underlie the excitatory and inhibitory learning deficits that we observed [31], [32], [50]. The demonstrated role of DA pathways in CI is consistent with broader theories of the role of DA in learning and specifically in mediating prediction error [32], [51]. Given the central role of DA systems in schizophrenia, prediction error processing in schizophrenia has been extensively investigated, largely in studies of blocking [52], in which a stimulus (B) is conditioned in the presence of a previously trained signal for that outcome (i.e. A+ followed by AB+). The outcome of B is already expected, and the resultant lack of prediction error thus curtails learning about B [48], [52], [53], [54], [55], [56], [57]. Participants with schizophrenia [48], [54], [55], [56], [57] “incorrectly” condition normally to the redundant cue, suggesting failure to compute the net prediction error to the AB compound. Relatedly, functional magnetic resonance imaging studies of human participants have shown that amphetamine increases the prediction error signal in striatal regions [58]. As learning about a conditioned inhibitor also depends on the correct assessment of net prediction error to a stimulus compound, signalling the absence of the expected outcome, a similar mechanism might underlie the CI deficit observed here.

However, to attribute effects on learning to an abnormality in prediction error processing is not always helpful: in simple conditioning prediction error does not depend on stimulus novelty - yet schizophrenic participants often show reduced conditioning to novel cues, but enhanced conditioning to a pre-exposed CS. A resolution to this paradox could lie in the suggestion made by some theories of learning, that prediction error is mediated through CS *associability* - ability to condition [59]. Stimuli that are pre-exposed, or followed by a predicted outcome (e.g., the added cue in a blocking experiment) lose associability, while stimuli followed by surprising outcomes gain associability, but then lose it as they become effective signals for that outcome. If the ease with which CS associability can change were impaired in schizophrenia, this could explain the observed pattern of results. In the blocking task, the added cue would lose less associability when paired with the predicted outcome, enhancing learning, while the increase in associability normally accruing to the conditioned inhibitor, on being paired with the unexpected absence of the outcome, would be curtailed, reducing CI.

More broadly, our understanding of the cognitive abnormalities accompanying schizophrenia could be advanced by an appreciation of the possible role of CI. For example, sensory flooding is frequently related to the aberrant assignment of salience - where an irrelevant cue is treated as significant [1], [3], [60], [61], [62], [63], [64], [65], [66], [67], [68]. Delusions could arise as the patient tries to make sense of aberrantly salient experiences [12], whereas hallucinations might reflect the anomalous salience of internal representations. Antipsychotic drugs, acting on DA D2 receptors, could dampen the salience of these abnormal experiences, alleviating symptoms [63], [64]. Reduced CI could be an additional mechanism through which salience is erroneously attributed: Inhibitory learning allows current environmental cues to signal that a stimulus which previously predicted an emotional event no longer does so. Impairment in such inhibitory learning would thus result in inappropriate responding to once valid predictors that are currently inoperative. Casually put, irrelevant cues would continue to be regarded as significant, and hence salient, thus contributing to the sensory flooding and delusional experiences characteristic of schizophrenia [1], [12], [67], [68].

Our results demonstrate for the first time that inhibitory learning is impaired in schizophrenia, and thus provide an attractive framework for interpreting the positive symptoms of schizophrenia. However, there was no evidence of any relationship between individual variation in CI and positive symptoms as measured by the PANSS - and participants with higher PANSS *negative* scores showed relatively greater impairment, in excitatory as well as inhibitory learning (although these data have their limitation). We acknowledge that the deficit in inhibition we observed could be related to an excitatory learning impairment at the training stage. Whatever its source, a better understanding of the relationship between CI and symptom profile may guide the development of better targeted cognitive-behavioural interventions for patients with schizophrenia. Accordingly the next step will be to investigate this relationship in a larger sample with two *a priori* defined sub-groups, displaying predominantly positive versus predominantly negative symptoms.

## Supporting Information

Supporting Information S1 **Supplementary statistics document: fully reported factorial analyses, both for the stage 1 excitatory training and the stage 2 inhibitory training.**
(DOCX)
