# Supplementary material for: When to Hold That Thought: An Experimental Study Showing Reduced Inhibition of Pre-trained Associations in Schizophrenia
Source: PLoS One. 2012 Jul 30;7(7):e42175. doi: 10.1371/journal.pone.0042175 (PMC3408477; doi:10.1371/journal.pone.0042175)
Supplement: Supporting Information S1 — Supplementary statistics document: fully reported factorial analyses, both for the stage 1 excitatory training and the stage 2 inhibitory training. (DOCX) [file pone.0042175.s001.docx]

**Supporting Information S1**

**Training stage 1: Excitatory training.** During the first training stage, the ratings of A and C steadily increased, while those to U and V fell gradually (Figure 2). This pattern of responding was observed in both groups. However, the ratings of A and C were lower in patients than in controls whilst the ratings of U and V were higher. Both of these observations suggest that the controls learned the discriminations better than the schizophrenic patients, and this impression was supported by the results of the statistical analysis.

ANOVA with group, discrimination (A versus U, and C versus V) and reinforcement as factors revealed no main effect of group or discrimination, both *F*s<1. However, the main effects of training block and reinforcement were significant, *F*(5,240)=2.31, *p=*0.05 and *F*(1,48)=260.66, *p<*0.001, *r*=.84, respectively. The interaction between reinforcement and diagnostic group was also significant, *F*(1,48)=7.73, *p=*0.008, *r*=.37 suggesting that performance on the discriminations differed in the two groups. Simple main effects analysis confirmed that the groups differed on both reinforced and non-reinforced trials, *F*(1,96)=4.40, *p=*0.04 and *F*(1,96)*=*7.95, *p=*0.006, respectively, suggesting that the schizophrenic group did not learn as well as the control group about either reinforced or non-reinforced trials. However, the difference between reinforced and non-reinforced trials was significant in both control and schizophrenic groups, *F*(1,48)=91.41, *p<*0.001, and *F*(1,48)=31.68, *p<*0.001, respectively, demonstrating that both groups nonetheless learnt the discriminations.

There was also an interaction between training block and reinforcement *F*(5,240)=17.92, *p*<0.001, *r*=.69 reflecting the development of the discrimination over training; simple main effects revealed that there was an effect of block for both reinforced and non-reinforced trials: *F*(5,480)=14.34, *p*<0.001; *F*(5,480)=7.68, p<0.001, and that the difference in ratings between reinforced and non-reinforced trials was significant on all training blocks. Consistent with the aforementioned difference between the diagnostic groups in the acquisition of the discrimination, there was also an interaction between training block, reinforcement and diagnostic group, *F*(5,240)=3.15, *p=*0.009, *r*=.17 Nothing else was significant, largest *F*(1,48)*=*3.14, *p=*0.08.

**Training stage 2: Inhibitory training.** During the second training stage, the ratings of AZ and CY showed some increase, while those to AP and BX decreased slightly. Figure 3B shows that overall participants learned the difference between the reinforced and non-reinforced compounds. Comparing the scores in the two groups, it can be seen that, just as in the previous stage, the ratings of AZ and CY were lower in patients than in controls, whereas the ratings of AP and BX were higher. Both of these observations suggest that the controls learned the discriminations better than the schizophrenic patients.

A mixed ANOVA with diagnostic group (schizophrenic patients v. controls) as between-subjects factor, and discrimination (AZ v. AP and BX v. CY), reinforcement (reinforced or not) and training block (1-2) as within-subjects factors, revealed no main effect of diagnostic group, discrimination or training block, largest *F*(1,48)=2.32, *p=*0.13. However, the main effect of reinforcement was significant, *F*(1,48)=106.06, *p<*0.001, *r*=.83, and this factor interacted significantly with diagnostic group, *F*(1,48)=11.08, *p=*0.002, *r*=.43; simple main effects analysis revealed that the groups differed on both reinforced and non-reinforced trials, *F*(1,96)=9.99, *p=*0.002 and *F*(1,96)*=*5.01, *p=*0.03, respectively, confirming the suggestion that the control group learned more effectively than the schizophrenic group; however, the difference between reinforced and non-reinforced trials was significant in both control and schizophrenic groups, *F*(1,48)=92.84, *p<*0.001, and *F*(1,48)=24.29, *p<*0.001, respectively, confirming that both groups had nonetheless learnt the discrimination.

As might be expected, there was a significant interaction between training block and reinforcement *F*(1,48)=11.12, *p=*0.002, *r*=.43. The simple main effects revealed that there was an effect of blocks for non-reinforced trials *F*(1,96)=12.17, *p*<0.001, but not for reinforced trials *F*(1,96)=2.04, *p*=0.16; the discrimination was nonetheless significant on both training blocks: *F*(1,96)=63.76, *p*<0.001; *F*(1,96)=115.64, *p*<0.001 respectively. A significant interaction was also found between discrimination and reinforcement *F*(1,48)=4.84, *p=*0.03, *r*=.30, suggesting that there might have been differences in the ease with which the AZ/AP and CY/BX discriminations were mastered; however, simple main effects revealed that ratings of AZ and CY, and AP and BX, did not differ *F*(1,96)=2.23, *p*=0.14, and *F*(1,96)=2.15, *p*=0.14 respectively, and that the discrimination was significant for both discrimination types, *F*(1,96)=59.67, *p*<0.001 for AZ and CY, and *F*(1,96)=99.50, *p*<0.001 for AP and BX, suggesting that both discriminations were learned effectively. Nothing else was significant, largest *F*(1,48)*=*2.47, *p=*0.12.
